# Tumor Selective Hyperthermia Induced by Short-Wave Capacitively-Coupled RF Electric-Fields

**DOI:** 10.1371/journal.pone.0068506

**Published:** 2013-07-04

**Authors:** Mustafa Raoof, Brandon T. Cisneros, Stuart J. Corr, Flavio Palalon, Steven A. Curley, Nadezhda V. Koshkina

**Affiliations:** 1 Department of Surgical Oncology, The University of Texas M. D. Anderson Cancer Center, Houston, Texas, United States of America; 2 Department of Surgery, The University of Arizona Health Science Center, Tucson, Arizona, United States of America; 3 Department of Chemistry, Rice University, Houston, Texas, United States of America; 4 Department of Mechanical Engineering and Materials Science, Rice University, Houston, Texas, United States of America; University of California, Irvine, United States of America

## Abstract

There is a renewed interest in developing high-intensity short wave capacitively-coupled radiofrequency (RF) electric-fields for nanoparticle-mediated tumor-targeted hyperthermia. However, the direct thermal effects of such high-intensity electric-fields (13.56 MHZ, 600 W) on normal and tumor tissues are not completely understood. In this study, we investigate the heating behavior and dielectric properties of normal mouse tissues and orthotopically-implanted human hepatocellular and pancreatic carcinoma xenografts. We note tumor-selective hyperthermia (relative to normal mouse tissues) in implanted xenografts that can be explained on the basis of differential dielectric properties. Furthermore, we demonstrate that repeated RF exposure of tumor-bearing mice can result in significant anti-tumor effects compared to control groups without detectable harm to normal mouse tissues.

## Introduction

Hyperthermia has been used in the treatment of cancer for centuries. There are reports from as early as 3000 BC of the application of heat for cancer treatment by ancient Egyptians [1]. The first reports from a formal scientific investigation of cancer hyperthermia came from Busch and Coley in the late 19^th^ century and described the disappearance of sarcomas after spontaneous fever secondary to erysipelas infection [2]. While treating cancer with hyperthermia is an age-old proposition, there are ongoing challenges to its application in the clinic.

It is well known now that certain cancer cells are more susceptible to hyperthermia than normal cells [3]. The majority of work in cancer hyperthermia has focused on this premise, but the mechanisms of cancer cell death after hyperthermia is an area of active investigation. Numerous studies indicate the ability of hyperthermia to enhance efficacy of standard anticancer chemotherapy [1]. In the1980s the synergy between hyperthermia and radiation was studied in clinical protocols. However, the initial enthusiasm subsided due to the lack of tumor selective hyperthermia delivery systems and difficulties in real-time temperature control [4]–[6]. Over the past decade, advances in nanotechnology, molecular biology, and cancer research have set the path for the development of much more effective, tumor selective, non-invasive nano-hyperthermia systems.

Hyperthermia has a narrow therapeutic index so heating tumors using whole body or direct contact hyperthermia is usually highly invasive, and frequently not practical or feasible because of thermal damage to systemic or local normal tissues [7], [8]. The majority of non-invasive hyperthermia systems that currently are under development use electromagnetic non-ionizing radiation such as light, near infrared (NIR), and radiowaves for thermal therapy [1], [9]. As the penetration depth of electromagnetic radiation is inversely related to the frequency of the incident electric field, the use of short-wave radiofrequencies (with penetration depths <30 cm) is favored over microwaves or infrared light, where the penetration depths are limited to 1–3 cm [10]. However, microwave and infrared systems have been used successfully for non-invasive superficial, interstitial, and endocavitary applications [11].

Delivering hyperthermia to deep-seated tumors in a non-invasive fashion continues to be a major challenge. We have previously described a capacitively coupled radiofrequency system (13.56 MHz), the Kanzius external RF generator, which we have used in our efforts to develop a safe and effective nano-hyperthermia system [12]–[18]. These proof-of-concept studies have demonstrated that radiowave activation of metallic or semiconducting nanomaterials within tumor tissue can generate thermal toxicity, but our understanding of solely interaction of the RF fields with tumor and normal tissues using this system (without nanoparticles) is incomplete.

The primary goal of the present study was to evaluate thermal energy deposition in various mouse tissues as well as orthotopically-implanted xenografts of human liver and pancreatic cancer using the Kanzius RF generator. In addition, we evaluated the dielectric properties of tumors and normal tissues to explain the physical basis of tumor-selective hyperthermia. Finally, we evaluate the anti-tumor effect of high-intensity, short duration RF-generated hyperthermia treatments in orthotopic mouse models of human cancer.

## Methods

### Cell Lines, Materials and Tissue Processing

Human cancer cell lines (Hep3B, HepG2, and Panc1) were purchased from the American Type Culture Collection (Manassas, VA) and maintained as per the instructions of the supplier. Human pancreatic adenocarcinoma cell line MDA PATC-3 was kindly provided by Dr. Logsdon (M.D. Anderson Cancer Center, Houston, TX). Cells were derived from resected pancreatic adenocarcinoma tissue with informed patient consent. MD Anderson Cancer Center Institutional Review Board approved the protocol for tumor tissue collection [19]. MEM (for HepG2 and Hep3B) or DMEM (for Panc1 and MDA PATC-3) was supplemented with 10% (v/v) fetal bovine serum. The media were additionally supplemented with sodium pyruvate, non-essential amino acids, Penicillin G, and Streptomycin. Cells were cultured in T-75 or T-150 tissue culture flasks (Corning Inc., Corning, NY). For each cell line, the short tandem repeat fingerprint was confirmed by the Cell Line Characterization Core Service (M. D. Anderson Cancer Center, Houston, TX) within one year of all experiments. All media and supplements were purchased from Gibco (Life Technologies, Grand Island, NY). The cells were passaged approximately every three to five days before reaching confluency. Media was replaced every three days.

### Orthotopic Tumor Models

For *in vivo* studies orthotopically implanted mouse models of human liver and pancreatic tumors were generated. Mouse models of human HCC were generated in C.B-17 SCID mice (Taconic, Hudson, NY). Mouse models of human pancreatic tumors were generated in SCID nude mice (NCI, Bethesda, MD). Female mice between 4–5 weeks in age were purchased and acclimatized in M.D. Anderson animal facilities for up to 1 week. This study was carried out in strict accordance with the recommendations in the Guide for the Care and Use of Laboratory Animals of the National Institutes of Health. The Institutional Animal Care and Use Committee (IACUC) of UT M.D. Anderson Cancer Center approved the protocol (Protocol Number: 020801432). All surgery was performed under isoflurane anesthesia. Buprenorphine was used for additional post-operative pain control. All RF experiments were performed under ketamine and xylocaine anesthesia. All efforts were made to minimize suffering.

For liver tumor models approximately 1.6 million cells (Hep3B or HepG2) were injected in 0.01 ml of PBS in the left lobe of the liver using a 30-gauge needle though a transverse incision in the abdomen after anesthetizing mice per standard protocol. Similarly, for pancreas tumor models, 1 million cells (Panc-1 or MDA PATC3) suspended in 0.05 ml matrigel were injected into the animal pancreas through a left lateral incision.

Three to four weeks after implantation of tumor cells in the liver, bioluminescence measurements were performed. Firefly D-luciferin (Caliper Life Sciences, Hopkinton, MA) was administered by intraperitoneal (i.p.) injection at a dose of 150 mg/kg in 0.1 ml. Animals were anesthetized using 2.5% isoflurane and imaged using a Xenogen system IVIS-200 (Caliper Life Sciences, Hopkinton, MA) 5 minutes after the injection. The imaging was performed over 2 minutes with a 1x1 binning. Mice that had any bioluminescence activity above background (suggesting the development of tumors) were included in the subsequent study. Based on bioluminescence, approximately 95–99% of mice develop tumors by 3 weeks after implantation of cells for both tumor models. For pancreatic tumors bioluminescence measurements were not necessary as 100% mice developed tumors after one week of tumor cell implantation.

### Radiofrequency Generator Setup

**Figure 1:**
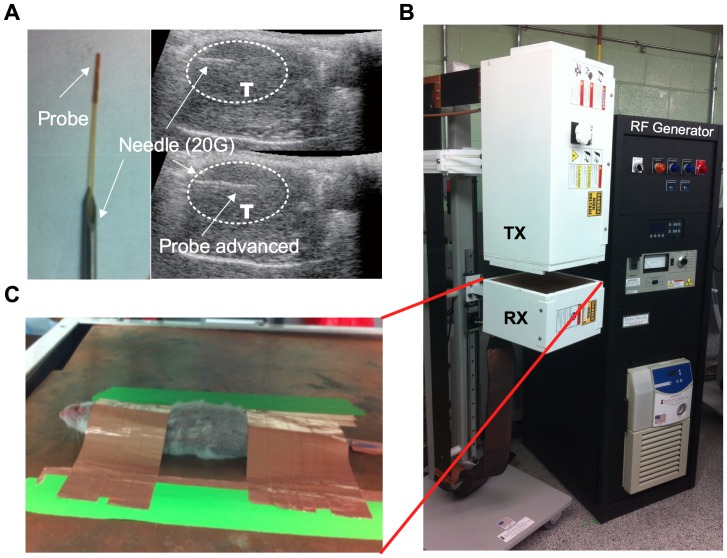
Radiofrequency generator and fiber optic probe placement. *Panel A.* For fiberoptic thermography, a temperature-sensing probe is placed through a 20 G needle. The needle is advanced into the tumor (T) under ultrasound guidance. The probe is then advanced through the needle and the needle is withdrawn. *Panel B.* Kanzius 13.56 MHz external RF generator system is shown (black box) that is connected to an end-firing antenna in the transmission head (Tx). A spacing of 3.5 inches exists between the Tx head and the receiver head (Rx)/ground plate. *Panel C.* A CB17 SCID mouse is placed supine on the ground plate of the Rx head. A copper shield made from copper tape is used to ground all mice and prevent electrothermal injury. An abdominal window is created in the middle of the copper shield to allow RF field exposure to the tumor-bearing area.

A Kanzius non-invasive external RF generator (ThermMed, LLC, Erie, PA) was used for animal hyperthermia exposures. The use of this generator has been described previously [16]. The generator operates at an adjustable output power (0–2 kW) at a fixed frequency of 13.56 MHz. The generator is connected to a high-Q coupling system with a Transmitter, i.e. Tx head (focused end-fired antenna circuit) and reciprocal, i.e Rx head (as a return for the generator) mounted on a swivel bracket allowing the RF field to be oriented in either a horizontal or vertical direction (Figure 1). The two heads were set at a distance of 3.5 inches apart. The Tx head is covered with a Teflon plate and the Rx head has a conducting copper surface to allow grounding of the animals as described later. The coaxial end-fire circuit in the Tx head produces a uniform RF electric field up to 15 cm in diameter. The field generated is predominantly electric with minimal magnetic component.

For animal RF field exposures, mice were anesthetized with a cocktail of ketamine (100 mg/kg i.p.) and xyalzine (10 mg/kg i.p.). Hair was removed from the anterior surface of the abdomen using clippers. Before RF exposures, certain steps were taken to ensure prevention of electrothermal injury. First, water access was blocked for 1 hr before treatment and urine was removed from the bladder by gently pressing on the lower abdomen after anesthesia. Secondly, mice were grounded with copper tape as shown in Figure 1. A window was created within the copper tape grounding-shield to allow RF exposure to the abdomen. All experiments were performed for 10 min at 600 W power output to be consistent with prior reports in the literature.

### Thermal Imaging and Fiber Optic Thermography

During RF-field exposure temperature from the abdominal surface of mice was recorded using an infrared thermal camera (FLIR SC 6000, FLIR Systems, Inc., Boston, MA). Fiber optic thermography was performed using Fluotemp, a fiber optic probe (PhotonControl, Burnaby, BC, Canada), 400 microns in diameter with a scientific accuracy of ±0.1°C. A 20 G, 1-inch needle was placed in the intended tissue under ultrasound guidance and the probe was advanced into the target organ (Figure 1). Subsequently, the needle was retracted over the fiber optic probe. This probe was pre-tested for lack of heating in the RF field. Real-time temperature recording was performed from both temperature-measuring devices and analyzed using LabVIEW. Thermal dose was calculated using the following equation [8], [20]:
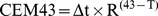



Where, CEM43 is thermal dose stated as cumulative equivalent minutes at 43°C, Δt is the time interval, T is the average temperature during Δt, R is a constant and is calculated to be 0.5 for T≥43°C and 0.25 for T<43°C.

### Histological Analysis

Tumor tissues harvested at the end of the experiment were fixed in 10% buffered formalin (pH 7.0) for 24 hours and subsequently stored in 70% (v/v) ethanol before embedding them in paraffin. For histological analysis 5-micron sections were placed on a glass slide and tissue sections were deparaffinized and rehydrated. The slides were stained with eosin and hematoxylin and a cover slip was sealed in place. Tissue slides of vital organs from mice after single and continuous RF exposure were analyzed by a dedicated veterinarian pathologist.

### Permittivity Measurements

Complex permittivity measurements were taken within 30 minutes of harvesting tissues from mice. Tumor and normal tissues were sharply dissected into small fragments and at least two fragments were analyzed from each tumor or organ. Measurements were obtained using an Agilent 85070E high-temperature coaxial dielectric probe (Agilent Technologies, Santa Clara, CA) connected to an Agilent E4991A impedance analyzer across the frequency range 10 MHz−3 GHz at room temperature. Approximately 800 logarithmic data points were taken across the specified frequency range with each measurement taken 3 times.

### Statistical Methods

Results are reported as mean ± SEM unless otherwise noted. For all inferential statistics, null hypothesis was rejected at p<0.05. Two-sided Student’s t-test was used for two-group means. Multiple group comparisons were performed using one-way ANOVA. All data was plotted and analyzed in Prism 5.0 (La Jolla, CA).

## Results

### Relationship of Electric Field and RF Input Power

**Figure 2:**
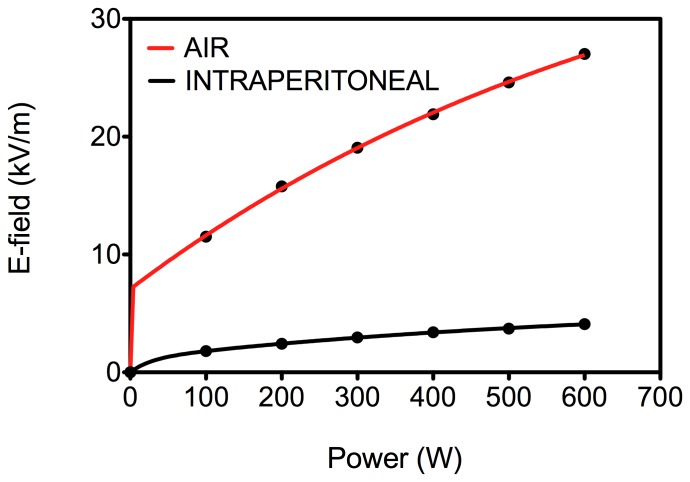
E-field measurements. Electric field measurements were performed using the setup described in methods. The measurements were performed in air or in the peritoneal cavity of mice while keeping the E-field probe at the same *x,y,z* location, which approximately corresponded with the location of orthotopic tumors in tumored mice. *(n = 3, data points represent mean, and error bars represent standard deviation).*

High-intensity electric-field (E-field) measurements are notoriously difficult because of the lack of reliable methods and equipment heating at such high E-field strengths. Nonetheless, the RF E-field determination was made using a custom-made 28 cm long E-field probe (designed by ThermMed, LLC, Inc., 332 Erie, PA) connected to an oscilloscope as previously reported [21]. Using this setup, it was possible to accurately measure the RF voltage up to 600 W RF power, at which point the probe started to heat significantly. The magnitude of E-field strength versus power (100−600 W) is shown in Figure 2. As shown, the E-field showed a linear power-dependent response across the measured power range. The first set of measurements were performed in air at the approximate location of the tumor in the E-field ∼1.5 cm from the Rx head. These measurements were then repeated in the peritoneal cavity of a mouse. As expected, the E-field demonstrates attenuation within the intraperitoneal cavity. Given the diameter of the E-field probe it was not possible to measure E-field strength in every tumor tissue or organ.

### Radiofrequency Exposure Results in Tumor-selective Hyperthermia

**Figure 3:**
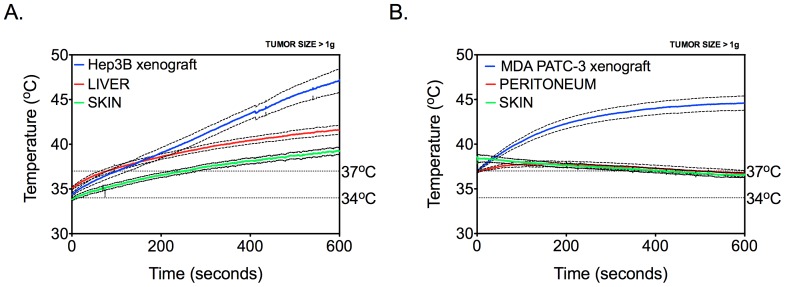
Thermal dose quantification in Hep3B and MDA PATC-3 xenografts under RF field exposure (13.56 MHz, 600 W). *Panel A.* Hep3B xenograft and normal mouse liver temperatures were measured in real-time using fiber optic thermography while abdominal surface/skin temperatures were measured using infrared thermography. RF exposure was started at a tumor temperature of 35°C. *(n = 6). Panel B.* MDA PATC-3 xenograft and intra-peritoneal temperatures were measured in real-time using fiber optic thermography while abdominal surface/skin temperatures were measured using infrared thermography. RF exposure was started at a tumor temperature of 37°C. *(n = 9). (Solid line represents mean, dashed line represents standard deviation, and vertical dotted line represents frequency of 13.56 MHz).*

Temperature changes in various mouse tissues and orthotopic liver and pancreatic tumor xenografts were recorded using a fiber optic thermal probe. The thermal probe was placed under sonographic guidance inside the studied tissue and real-time temperature measurements were performed during the 10 min of RF exposure. Skin temperature was measured using an infrared thermal camera. The results are demonstrated in Figure 3. As shown, the temperature of orthotopically implanted human Hep3B hepatocellular carcinoma xenografts increased at a higher rate compared to the normal mouse liver and achieved the average level of 47±2°C. We also noticed a 2–3°C increase in skin surface temperature compared to the orthotopic liver tumor. Thermal dose by convention is expressed in cumulative equivalent minutes at 43°C as CEM43 as described in the Methods section. The average calculated CEM43 values for liver tumor and normal liver was 1041.6 and 17.5, respectively. These findings demonstrate tumor-selective hyperthermia delivered by RF field exposure to mice bearing human liver cancer xenografts in situ. We then evaluated radiofrequency heating of orthotopic pancreatic cancer, MDA PATC-3, human xenografts. Since it was not possible to reliably place the temperature probe in the normal pancreas, intraperitoneal temperature measurements were used instead. The average temperature in pancreatic tumors was lower (p<0.01) when compared with orthotopic liver tumors and achieved 44±2°C, but it was significantly higher (p<0.01) than the temperature in the abdominal cavity and that of the body surface, which remained within physiological range and did not significantly exceed 37°C. This resulted in an average calculated CEM43 of 46.5 in pancreatic cancer after 10 min of RF exposure. In fact, we noted a slight decline in surface and peritoneal temperatures with time. We believe that temperature decrease in the peritoneal cavity and the skin is due to inhibition of thermoregulation in mice after ketamine anesthesia. While ketamine has been shown to be thermoregulatory sparing, mild inhibition still occurs [22]. Due to loss of heat conservation the animal loses heat to ambient environment that is at a lower temperature compared to starting core body temperature of the mouse. In contrast skin temperature rise in liver tumor models most likely is a result of heat transfer from liver and liver tumor xenograft to the skin by virtue of its close proximity which is not seen with deeper pancreatic cancer xenografts.

### Tumors have Higher Dielectric Losses Compared to Normal Tissues

Radiowave energy absorption and heat generation within biologic tissues is dependent on their dielectric properties. The ability of a material to store and dissipate electrical energy as heat can be described by the real (ε′) and imaginary (ε″) parts of the complex permittivity function (ε*). This relationship is given by equation 1:



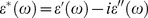
(1)


Where, ω is the radial frequency (2πf). Equation 1 can be modified to give a direct relationship to specific absorption rate (SAR, W/kg) with respect to electric-field strength (E) and permittivity (ε″)/conductivity (σ) according to equation 2:
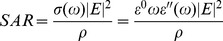
(2)


**Figure 4:**
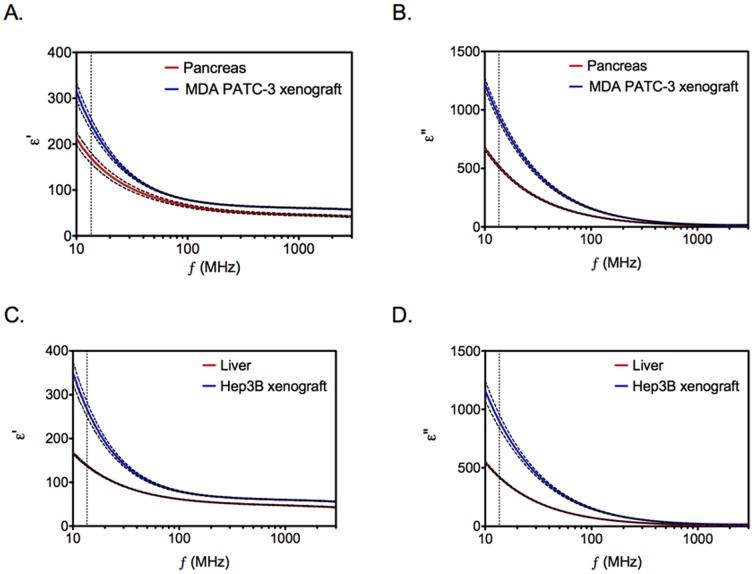
Dielectric properties of tumor tissues. *Ex-vivo* dielectric spectroscopy of tumors (Hep3B and MDA PATC-3 xenografts) and normal tissues (liver and pancreas) was performed. Permittivity (ε’) and imaginary permittivity (ε”) are shown for each tissue. *(n = 4–15, Solid line represents mean, dashed line represents standard deviation, and vertical dotted line represents frequency of 13.56 MHz). To compare permittivity values of normal and tumor tissues, two-tailed unpaired Student’s t-test was performed. p<0.01 for all datasets.*

**Figure 5:**
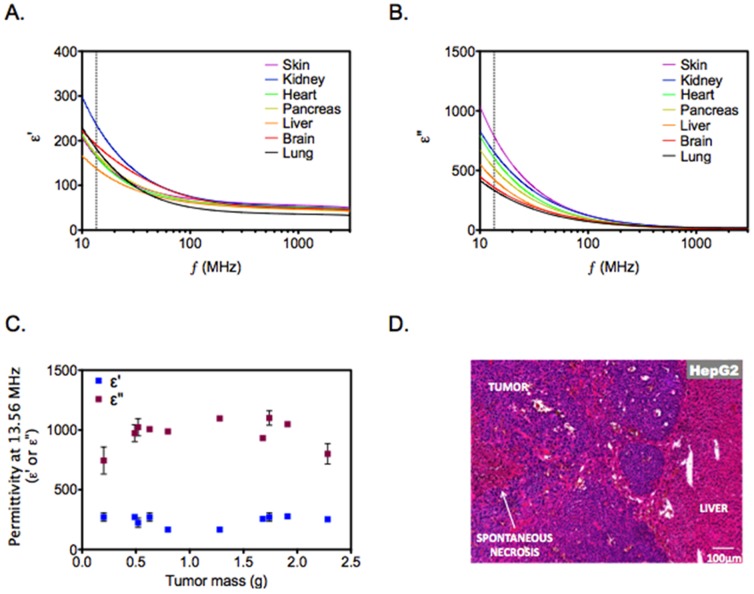
Dielectric properties of normal mouse tissues. *Panel A, B. Ex-vivo* dielectric spectroscopy of normal mouse tissues was performed. Permittivity (ε’) and imaginary permittivity (ε”) are shown for each tissue. *(n = 3–15, Solid line represents mean, dashed line represents standard deviation, and vertical dotted line represents frequency of 13.56 MHz)). Panel C.* Variation of tumor dielectric properties with respect to tumor mass. *The* permittivity (ε’) and imaginary permittivity (ε”) values of MDA PATC-3 xenografts with variable mass are shown at 13.56 MHz. *(n = 3–6, data points represent mean, and error bars represent standard deviation). Panel D.* Areas of spontaneous necrosis were seen in untreated orthotopic xenografts, which were not seen in adjacent normal liver or pancreas. A representative figure of a HepG2 xenograft is shown at the tumor margin. Similar observations were noted for Hep3B, Panc1 and MDA PATC-3 xenografts *(not shown)*.

Where, ε^0^ is the vacuum permittivity of free space (8.85 × 10^−12^ F/m), ω is the radial frequency, and ρ is the density (kg/m^3^). Since the tumor and normal tissue densities are known to be similar, we hypothesized that the observed heating behavior could be predominantly explained by increased energy deposition in tumors as a result of higher imaginary permittivity (ε”) in tumors compared to normal tissues. For that purpose, we performed *ex vivo* measurements of dielectric properties of normal and tumor tissues. The results shown in Figure 4 demonstrate significantly higher ε′ and ε″ values for liver and pancreatic tumors compared to normal liver and pancreas, respectively. Next, we evaluated the dielectric properties of normal tissues and the dispersions are presented in Figure 5. We note that with the exception of skin/subcutaneous tissues, normal tissues have lower real and imaginary permittivity values than tumor tissues. We also note very little variation in these parameters between tumors of different mass suggesting that SAR across tumors of variable size should be similar. It has been previously reported that necrotic tissues have higher conductivity, which may explain higher SAR values in these tissues when exposed to the RF field [23]. Histological examination of orthotopic tumors demonstrated variable degree of spontaneous necrosis. This was not observed with normal mouse liver and pancreas.

### Tumor Response to RF Hyperthermia

**Figure 6:**
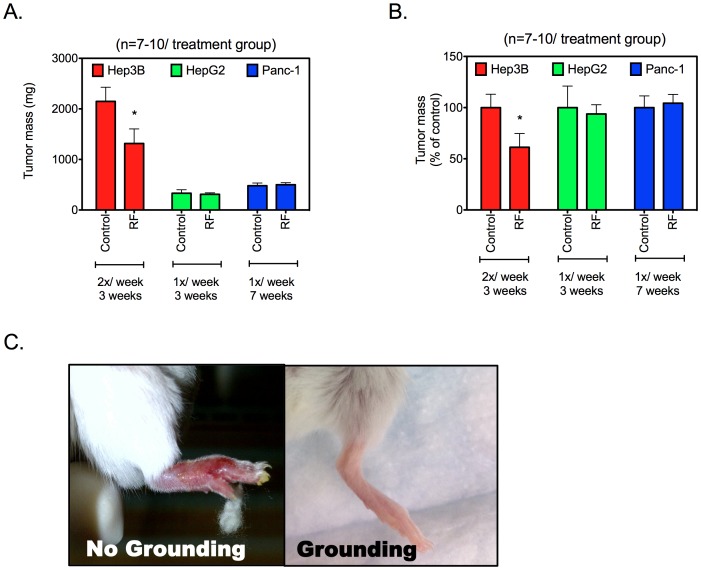
Effect of multiple RF exposures on xenografts and normal tissues. *Panel A.* Orthotopic tumor-bearing mice were exposed to RF multiple times as shown. Tumor mass was measured at the end of experiment. Each treatment consistent of a 10 min RF field exposure at 600 W, 13.56 MHz. Treatment repetition frequency and total number of treatments were arbitrarily chosen based on the growth of the tumor model. Tumor mass is represented for treated and untreated groups. (n = 7–10/group, bars represent mean, and error bars represent standard deviation, * p<0.01, two-tailed unpaired Student’s t-test). Panel B. Data is normalized to the average tumor mass of each control group for relative comparison. *Panel C.* Protective effects of copper grounding are demonstrated. Deep partial thickness burns are noted on the foot of a mouse that was not grounded 48 hours after a 10 min RF exposure *(Left*). Copper tape grounding was successful in preventing these burns *(Right).*

After demonstrating administration of tumor-selective hyperthermia using a short-wave capacitive RF field, we wanted to evaluate the long-term consequences of repeated high-intensity RF exposure on murine tissues and implanted xenografts of human liver and pancreatic cancers. For that purpose we utilized three different animal models that demonstrate variable tumor growth rates. Frequency and total number of RF treatments was varied based on the growth rate of tumors. For instance, fast growing hepatocellular carcinoma Hep3B xenografts were treated twice a week for three weeks whereas slow growing hepatocellular carcinoma HepG2 xenografts were treated once a week for three weeks. We treated Panc-1 tumors once a week for seven weeks considering the relatively slow growing nature of these tumors. The effect of RF exposure on tumor mass was studied in comparison to untreated controls (Figure 6). As shown, Hep3B tumors exposed to RF treatments were significantly smaller compared to untreated controls (p<0.05). However, we note that RF-exposure did not have a significant effect on Panc-1 or HepG2 xenografts. Histological analysis of tumors to evaluate necrosis did not demonstrate any significant differences between RF exposed and untreated controls for any of the tumor models examined.

### Adverse Effects of RF Exposure in Mice

Several precautions were necessary to prevent adverse events in mice exposed to high intensity short duration RF fields. In our experience, it is essential to ground the mice to the Rx head by using copper tape. This minimizes the majority of surface electrical burns. Figure 6C demonstrates manifestations of thermal injuries in mice that may occur in mice without grounding. Body parts with pointed geometries such as the tail, forelimbs and hind limbs were more frequently injured. Signs of thermal injury do not manifest until several hours after RF exposure. After waking up from anesthesia mice who suffer thermal injuries typically demonstrate a hunched posturing and seclusion in the corner of the cage with limited movement. Mild erythema can be appreciated a few hours later. This gradually evolves into swelling and subsequent skin necrosis. These side effects were successfully prevented by electrically grounding the mice by covering each animal’s tail, ears, and limbs with copper tape attached to the copper plate on which the animals were placed. Occasionally, burns in the urogenital area were noted in association with urination during RF exposure. These were successfully prevented by fluid restriction and bladder emptying prior to RF exposure.

We note that with proper precautions, all of the adverse events could be eliminated. Mice were observed for behavioral changes. We noted decreased grooming in mice treated with RF for the 24 hours after RF exposure followed by a return to baseline behavior thereafter. Gross and histological analysis of mouse tissues also failed to demonstrate any evidence of tissue injury or necrosis suggesting that the RF treatments were well tolerated when appropriate precautions were taken.

## Discussion

In this manuscript we have presented a systematic study of electrical properties of human liver and pancreatic orthotopic cancer xenografts and normal mouse tissues. We have also studied the heating of these tissues in mouse models when exposed to RF E-Fields using a non-invasive RF generator. Finally we have investigated the long-term anti-tumor and adverse effects of RF E-fields in several mouse models of human liver and pancreatic cancer.

The Kanzius RF generator has been under scrutiny for several years as a promising treatment modality to treat advanced chemoresistant solid tumor malignancies [12]–[17]. The premise of this therapy involves RF heating of metallic nanoparticles targeted to cancer cells. This method has been put to test in pre-clinical mouse models previously with encouraging results [16]. The studies described herein were designed to advance our understanding of the heating effects of RF field on murine cancer models without introduction of metallic nanoparticles. High-intensity, brief duration, short-wave RF fields were employed for the purposes of the study. Unlike prior studies [15], [16], the E-field was better characterized using a custom built and validated probe. At such high E-field intensities, thermography of mouse internal organs and orthotopic tumors presents a significant challenge. Conventional thermocouples interfere with the E-field and heat substantially [24]–[26]. This was overcome by using a non-metallic fluorescence-based temperature fiber-optic probe that does not heat or interfere with the high-intensity E-field. This allowed for real-time thermography during RF exposures.

In order to model human liver and pancreatic cancers, tumor cells were implanted in orthotopic locations. When tumor-bearing mice were exposed to the RF field, we noted significantly higher heating in tumors in comparison to normal mouse viscera. Selective solid tumor heating has been reported previously in subcutaneous tumor xenografts, however, comparative data on electrical properties of tumor and normal tissues was not reported [24]. We demonstrate herein that the measured dielectric properties can help explain tumor selective heating. We note that liver tumors have higher imaginary permittivity in the MHz-range compared to normal liver. Smith *et al.* also noted these differences between VX2-liver tumors and normal rabbit liver [27]. In addition we demonstrate similar permittivity differences between pancreatic tumors and normal pancreas. Values of ε’ and ε” were lower for pancreatic tumors when compared with liver tumors, indicating that pancreatic tumors are not able to store and dissipate electrical energy as much as liver cancer. This explains why the temperature inside hepatic tumors was higher than that for pancreatic tumors during the RF exposure. We further note that the size of tumor did not have an impact on its dielectric properties suggesting effective RF energy deposition irrespective of size. The differences in ε” values can be explained on the basis of biological composition of tissues. For instance, previous studies have demonstrated that degree of tissue necrosis strongly correlates with imaginary permittivity at low RF frequencies [23]. Cell necrosis leads to loss of cell membranes and the associated impedance between the intra and extracellular compartments. Therefore, at low radiofrequencies electrical conductivity increases in necrotic tissue that results in higher imaginary permittivity. Consistent with these observations, we noted evidence of necrosis in untreated liver and pancreatic tumors, which was not seen in normal liver and pancreas. It is plausible that dielectric properties of tumors could be predicted by evaluating degree of necrosis in various tumors. It is also possible that there is significant variation in tumor selective hyperthermia based on the degree of necrosis. However, these hypotheses remain to be tested in future work.

While we demonstrate successful delivery of hyperthermia using the non-invasive Kanzius RF generator in pancreatic and liver tumors, we were unable to demonstrate anti-tumor effect in all tumor models. It is important to note that hyperthermia was successfully achieved in tumors weighing more than 1 g. It was not possible to reliably place a thermography probe in smaller tumors and therefore the size dependency of RF heating could not be studied in mouse models. We note that fast growing Hep3B xenografts were most responsive to RF treatments. These tumors were also larger in size compared to slow growing HepG2 and Panc1 xenografts.

The lack of anticancer response of RF treatment in Panc-1 and HepG2 tumors could be explained by the lower dose of therapy: mice with Panc-1 and HepG2 received treatment once weekly in contrast to Hep3B that received twice weekly treatments. Another obvious reason could be the difference in thermotolerance between these cancer cells, though our *in vitro* studies showed that Hep3B and HepG2 cancer cells showed similar sensitivity to the RF treatment (Fig. S1). This indicates on other factors that may contribute to differential tumor response in various tumor models using RF treatment such as tumor size, tumor vascularity, and blood flow. Future studies are already underway and will address these possibilities. Skin and subcutaneous tissue heating has been reported to be a significant problem in other short-wave capacitive RF-field generators [24], [26]. This problem was also encountered in our pilot experiments as well. We observed that skin and subcutaneous tissues have high imaginary permittivity compared to other normal mouse organs and therefore explains its higher heating in the E-field. However, applying copper tape to ground the animals successfully provided a simple solution and prevented thermal injury to skin in all the mice subsequently treated. Detailed histological analysis of other mouse tissues also failed to demonstrate toxicity to these tissues after RF exposures that achieve clinical hyperthermia in the mouse tumors. The shielding of undesired areas of the body from RF-induced hyperthermia via copper grounding tape can be used in future to avoid damage of normal areas in the body and increase the safety of the treatment. Practicality and effectiveness of this grounding method is currently being studied in our laboratory using larger porcine models. However, because of the larger surface area of a large animal like pigs, the dispersive area of the RF E-energy should reduce the risk of thermal damage to the skin and permit use of standard grounding pads similar to those used for electrocautery applications during surgical procedures.

In conclusion, tumor selective heating property of shortwave RF treatments causes negligible damage to normal tissues and, therefore, has a clear advantage over non-selective hyperthermia systems. Numerous publications indicate the utility of combining hyperthermia with chemotherapy and radiation for cancer treatment. We therefore anticipate that tumor selective hyperthermia systems, such as the one reported in this study, will be of great benefit as adjuncts to conventional cancer therapeutics in the near future.

## Supporting Information

Figure S1 **Cytotoxic effect of RF treatment on hepatocellular carcinoma cells in vitro.** Exponentially-growing adherent monolayers of Hep3B and HepG2 cells were exposed to the varying duration of RF exposure in a 12-well plate. The Kanzius RF generator set-up for in vitro studies has been described previously [14]. Viability was measured using a standard MTT assay as a percentage of untreated controls 24 hours after RF exposure.
(PDF)
