# Supplementary figures and images for: Tumor Selective Hyperthermia Induced by Short-Wave Capacitively-Coupled RF Electric-Fields
Source: PLoS One. 2013 Jul 4;8(7):e68506. doi: 10.1371/journal.pone.0068506 (PMC3701653; doi:10.1371/journal.pone.0068506)

FIGURE S1

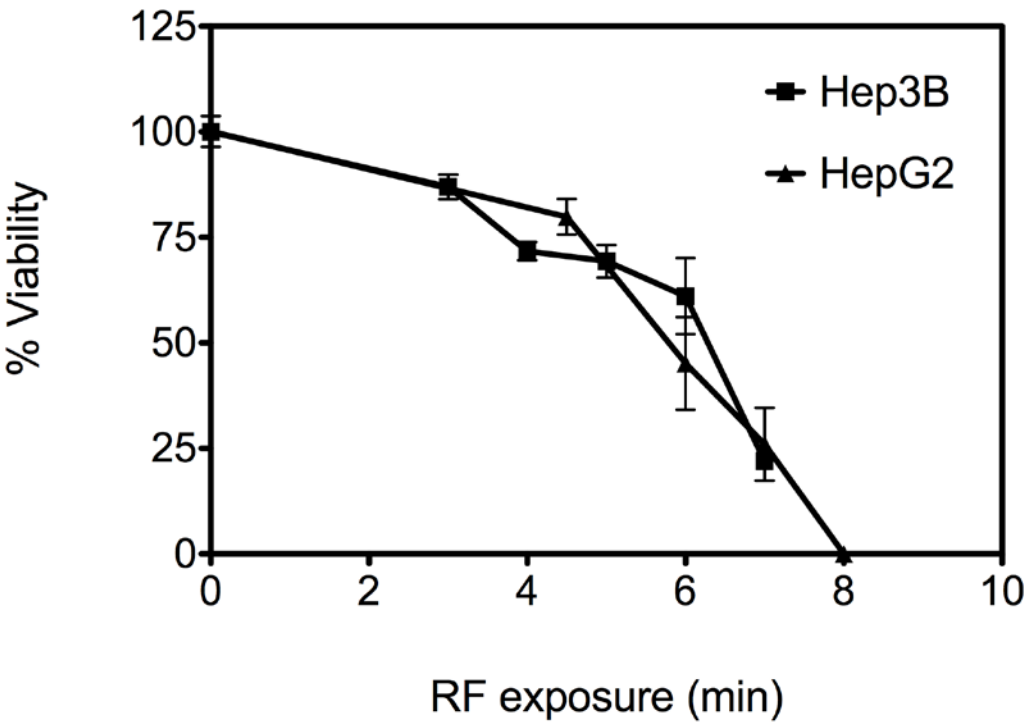

Supplement: Figure S1 — Cytotoxic effect of RF treatment on hepatocellular carcinoma cells in vitro. Exponentially-growing adherent monolayers of Hep3B and HepG2 cells were exposed to the varying duration of RF exposure in a 12-well plate. The Kanzius RF generator set-up for in vitro studies has been described previously [14]. Viability was measured using a standard MTT assay as a percentage of untreated controls 24 hours after RF exposure. (PDF) [file pone.0068506.s001.pdf]
